# Effect of traditional Chinese medicine combined with conventional Western medicine for patients with severe/very severe chronic obstructive pulmonary disease: a multi-center, randomized, double-blind, controlled study

**DOI:** 10.1186/s13020-025-01117-x

**Published:** 2025-05-20

**Authors:** Jiansheng Li, Yang Xie, Minghang Wang, Suyun Li, Xuefeng Yu, Nianzhi Zhang, Zhengang Zhu, Wei Zhang, Jihong Feng, Zikai Sun, Lin Lin, Zhijia Sun, Hailong Zhang, Xueqing Yu

**Affiliations:** 1 Co-construction Collaborative Innovation Center for Chinese Medicine and Respiratory Diseases by Henan & Education Ministry of P.R. China, Henan University of Chinese Medicine, No. 156 Jin-shui East Road, Zhengzhou, 450046 Henan People’s Republic of China; 2 Henan Key Laboratory of Chinese Medicine for Respiratory Disease, Henan University of Chinese Medicine, Zhengzhou, 450046 Henan People’s Republic of China; 3 Department of Respiratory Diseases, The First Affiliated Hospital of Henan University of Chinese Medicine, Zhengzhou, 450000 Henan People’s Republic of China; 4 Henan International Joint Laboratory of Evidence-Based Evaluation for Respiratory Diseases, Henan Province Clinical Research Center for Respiratory Diseases, The First Affiliated Hospital of Henan University of Chinese Medicine, Zhengzhou, Henan People’s Republic of China; 5 Department of Respiratory Diseases, The Second Affiliated Hospital of Liaoning University of Traditional Chinese Medicine, Shenyang, 110034 Liaoning People’s Republic of China; 6 Department of Respiratory, The First Affiliated Hospital of Anhui University of Traditional Chinese Medicine, Hefei, Anhui People’s Republic of China; 7 Department of Respiratory, The First Teaching Hospital of Tianjin University of Traditional Chinese Medicine, Tianjin, People’s Republic of China; 8 Department of Respiratory, Shanghai Shuguang Hospital Affiliated with Shanghai University of Traditional Chinese Medicine, Shanghai, People’s Republic of China; 9 Department of Respiratory, The Second Teaching Hospital of Tianjin University of Traditional Chinese Medicine, Tianjin, People’s Republic of China; 10 Department of Respiratory, Jiangsu Provincial Hospital of Traditional Chinese Medicine, Nanjing, People’s Republic of China; 11 Department of Respiratory, Guangdong Provincial Hospital of Chinese Medicine, Guangzhou, People’s Republic of China; 12 Department of Respiratory, The First Affiliated Hospital of Guangzhou University of Traditional Chinese Medicine, Guangzhou, People’s Republic of China

**Keywords:** Chronic obstructive pulmonary disease, Clinical trial, Randomised controlled trial, Traditional Chinese medicine

## Abstract

**Background:**

Traditional Chinese medicine (TCM) is widely used in the management of chronic obstructive pulmonary disease (COPD). This study aimed to evaluate the clinical efficacy of comprehensive therapy based on TCM patterns for patients with stable, severe to very severe COPD.

**Methods:**

A multicenter, randomised, double-blind, placebo-controlled trial was conducted. Eligible patients were randomly allocated in equal proportions to two groups: the trial group, which received TCM-based therapy with Bu-Fei Jian-Pi, and Bu-Fei Yi-Shen, and Yi-Qi Zi-Shen granules tailored to TCM syndromes, and the control group, which received a placebo resembling Chinese medicine. Both groups also received conventional Western medicine as part of their treatment. Acute exacerbations (AEs), lung function, dyspnea scores, the 6-min walking test (6MWT), and the COPD assessment test (CAT) were assessed over 12 months of treatment, with an additional 12 months of follow-up.

**Results:**

A total of 467 patients were included in the analysis with 228 in the experimental group and 239 in the control group. The Chinese herbal granules group significantly reduced AEs (0.63 vs. 1.03 events, *P* = 0.002), improved mMRC scores (−0.17 points, 95% CI −0.30 to −0.03; *P* = 0.015), 6MWT (29.24 m, 95% CI 10.71–47.77; *P* = 0.002), and CAT (−3.11 points, 95% CI −4.13 to −2.09, *P* < 0.001), compared with the control group. No significant differences were observed between the two groups in terms of FVC (l) and FEV_1_ (both in litres and as percentage).

**Conclusion:**

Comprehensive therapy based on TCM patterns demonstrated efficacy in patients with severe to very severe COPD, reducing the frequency of AEs, improving dyspnea and exercise capacity, and alleviating symptoms.

*Trial registration*: ClinicalTrials.gov, NCT02270424. Registered 17 October 2014, https://clinicaltrials.gov/study/NCT02270424?id=NCT02270424&rank=1.

**Supplementary Information:**

The online version contains supplementary material available at 10.1186/s13020-025-01117-x.

## Background

Chronic Obstructive Pulmonary Disease (COPD) is a common chronic airway disorder characterized by persistent respiratory symptoms and airflow limitation. Clinically, it is manifested by chronic cough, sputum production, and progressively worsening dyspnea. In advanced stages, COPD is commonly associated with frequent acute exacerbations (AEs) and chronic respiratory failure, severely affecting the quality of life and leading to a poor prognosis [1].

The global prevalence and mortality rates of COPD remain high. Among individuals aged over 40, the worldwide prevalence is 11.7% [2], while in China, it reaches 13.7%, affecting an estimated 100 million individuals [3]. COPD also imposes a substantial economic burden on patients. Studies show that the annual per capita hospitalization cost in the United States is $6852 [4], while in China, it ranges from $1964 to $3449 per year, accounting for 33–40% of a household’s average yearly income [5]. Although the disease-related burden has declined over the past three decades, COPD continues to present a major public health challenge in China [6].

In recent decades, significant advancements have been achieved in COPD research. While the onset and progression of the disease have been moderately slowed [7], many patients, especially those with severe or advanced stages, continue to experience rapid deterioration. AEs remain critical events that accelerate declines in lung function, increase hospitalizations, and worsen outcomes, including higher mortality rates [8–10]. Thus, reducing the frequency of AEs is a key goal in managing severe and very severe COPD.

Chronic inflammation plays a central role in the development of COPD, making glucocorticoids a key therapeutic option. However, oral glucocorticoids are associated with significant adverse effects, including osteoporosis, pneumonia, and metabolic disorders such as Cushing’s syndrome, glucose metabolism disturbances, and electrolyte imbalances [11]. Furthermore, their use may even increase mortality among stable COPD patients [12]. Consequently, inhaled corticosteroids (ICS) have become the preferred treatment. Long-acting β2-agonists (LABA) and long-acting muscarinic antagonists (LAMA), either alone or in combination, are also essential for COPD treatment. Triple therapy, which combines ICS, LABA, and LAMA, effectively improves lung function in severe and very severe cases [13]. However, evidence suggests that this therapy does not significantly reduce the frequency of AEs or mortality, and the overall prognosis may remain unchanged [14, 15]. Therefore, there is a pressing need for new treatment strategies that can improve efficacy and enhance patient outcomes in COPD management.

Traditional Chinese Medicine (TCM), supported by extensive clinical practice and experience, has been demonstrated to be effective in managing COPD by alleviating symptoms, reducing adverse events, and improving quality of life [16, 17]. Given the clinical characteristics of patients with severe and very severe COPD, our research team identified three predominant TCM patterns in these patients: Lung-Spleen Qi Deficiency, Lung-Kidney Qi Deficiency, and Lung-Kidney Qi and Yin Deficiency [18]. A comprehensive therapy based on these TCM patterns has been developed, emphasizing individualized treatment and refined through clinical practice and foundational research.

Studies have confirmed that this therapy can reduce the frequency and duration of AEs in moderate to severe stable COPD, alleviate symptoms, and enhance exercise capacity and quality of life [19, 20]. However, long-term studies evaluating the effectiveness and safety of this comprehensive therapy in severe and very severe COPD are still scarce. This study aims to fill these knowledge gaps and represents the first long-duration randomized controlled trial based on TCM theory for the treatment of severe to very severe COPD.

## Methods

### Trial design

This study is a multi-center, randomized, double-blind, placebo-controlled clinical trial involving ten hospitals (Table S1). It was approved by the Ethical Research Committees of the First Affiliated Hospital of Henan University of Chinese Medicine (2014HL-022-01) and registered in ClinicalTrials.gov (NCT02270424). The study was conducted in strict accordance with the approved protocol and the principles outlined in the Declaration of Helsinki.

### Participants

#### Diagnostic criteria

The diagnosis of COPD is based on the *Global Strategy for the Diagnosis, Management, and Prevention of Chronic Obstructive Lung Disease (GOLD 2013)* [21] and the *Chinese Treatment Guidelines of COPD* [22].

#### Diagnostic criteria in TCM

The Diagnostic Criteria for TCM Symptoms of COPD follow the Diagnostic Criteria for Chinese Medicine Symptoms of Chronic Obstructive Pulmonary Disease (2011), issued by the Pulmonary Diseases Specialized Committee of the Internal Medicine Branch of the Chinese Society of Traditional Chinese Medicine [23]. The detailed diagnostic criteria for TCM syndromes in COPD are outlined in Table S2.

#### Inclusion criteria

The inclusion criteria are as follows: (1) patients with stable COPD classified as grade 3 or 4 based on lung function; (2) age between 40 and 80 years; (3) Diagnosis of one of the following TCM syndromes: Lung-Spleen Qi Deficiency, Lung-Kidney Qi Deficiency, or Lung-Kidney Qi and Yin Deficiency; (4) a 2-week washout period prior to randomization; (5) No participation in other interventional trials within the previous month; (6) Signed informed consent form.

#### Exclusion criteria

Patients were excluded if one of the following criteria was met: (1) pregnant or breastfeeding women; (2) any psychiatric condition that impaired the patient’s ability to understand the study’s nature, scope, and potential consequences; (3) current respiratory disease other than COPD (e.g., bronchiectasis, bronchial asthma, tuberculosis, pulmonary fibrosis, pulmonary thromboembolic, diffuse pan bronchiolitis); (4) comorbid neural muscle disease affecting respiratory function; (5) comorbid heart failure (NYHA Class Ill or IV), or myocardial infarction within 6 months, or hemodynamic instability; (6) comorbid malignancy, congenital or acquired immunodeficiency; (7) Severe liver or renal disease (e.g., cirrhosis, portal hypertension, variceal hemorrhage, dialysis, or renal transplantation); (8) Participation in other clinical trials or known allergies to the medications used in the study.

### Interventions

The conventional Western medicine treatment followed *the Global Initiative for Chronic Obstructive Lung Disease (GOLD 2013)* [21] and *Chinese Treatment Guidelines of COPD* [22]. Severe and very severe COPD patients received Salmeterol/fluticasone for 52 weeks: Salmeterol/fluticasone (Seretide^®^, GlaxoSmithKline), 50/500 μg/dose, 60 inhalations. 50/500 μg each time, twice daily.

In addition to conventional Western medicine treatment, both the trial group and control group prescribed additional Chinese medicine granules and placebo according to the different TCM patterns. The granules and placebos, including Bu-Fei Jian-Pi, Bu-Fei Yi-Shen, and Yi-Qi Zi-Shen, corresponded sequentially to the TCM syndromes of Lung-Spleen Qi Deficiency, Lung-Kidney Qi Deficiency, and Lung-Kidney Qi and Yin Deficiency.

The TCM granules were compound preparations, with their components listed in Table 1. These components were produced and packed by Jiang Yin Tian Jiang Pharmaceutical Co. Ltd. with the authentication quality of Goods Manufacturing Practice, Jiangsu, PR China. The quality of drug batches met required standards. The TCM placebos consisted primarily of lactose, maltodextrin, and edible colorings, and were designed to resemble the test drugs in color, odor, taste, appearance, and weight. The packaging of the placebos was identical to that of the active drugs in the trial. Specific ingredient ratios and details are provided in Table S3.

**Table 1:** Main components of TCM granules

Chinese name	Latin name	Amount (g)
*Bu-Fei Jian-Pi granules*		
Huang Qi	*Astragalus propinquus*	15
Dang Shen	*Codonopsis pilosula*	15
Bai Zhu	*Atractylodes macrocephala*	12
Fu Ling	*Wolfiporia extensa*	12
Zhe Bei Mu	*Fritillaria thunbergii*	9
*Bu-Fei Yi-Shen granules*		
Ren Shen	Radix Ginseng	6
Huang Qi	*Astragalus propinquus*	15
Gou Qi Zi	*Lycium barbarum*	12
Shan Zhu Yu	*Cornus officinalis*	12
Yin Yang Huo	*Epimedium brevicornu*	9
*Yi-Qi Zi-Shen granules*		
Tai Zi Shen	*Radix pseudostellariae*	10
Huang Qi	*Astragalus propinquus*	10
Shu Di Huang	*Rehmannia glutinosa*	10
Mai Dong	*Ophiopogon japonicus*	10
Wu Wei Zi	*Schisandra chinensis*	6

The batch numbers for the granules are as follows:

- Bu-Fei Jian-Pi granules: Batch number 1402332, 5.82 g per bag
- Bu-Fei Yi-Shen granules: Batch number 1402333, 5.33 g per bag
- Yi-Qi Zi-Shen granules: Batch number 1402334, 5.37 g per bag

Each granule type was given orally, three bags each time, twice daily for a 52-week treatment period.

### Outcomes

#### Primary outcome measure

The primary outcome measure is the frequency of AEs in COPD. An AE is defined as an acute onset of respiratory symptoms in patients with chronic obstructive pulmonary disease, which are exacerbated beyond the normal daily variation (typically manifested by worsening dyspnea, cough, increased sputum volume, and/or purulent sputum), leading to the need for a change in drug therapy [24]. The frequency and duration of each AE were recorded at 52 weeks of treatment and 52 weeks of follow-up. If the interval between two AEs was less than 1 week, they were considered a single acute exacerbation.

#### Secondary outcome measures

1. Mortality: The mortality rate from COPD and all-cause mortality will be calculated.
2. Lung function: The indicators of forced vital capacity (FVC), forced expiratory volume in one second (FEV_1_), and FEV_1_ percentage of the predicted value will be assessed.
3. Dyspnea: The level of dyspnea will be assessed using the modified Medical Research Council (mMRC) scale [25, 26], a simple grading system that ranges from 0 (less severe) to 4 (severe).
4. 6 Minutes Walking Distance Test (6MWT): The 6MWT [27] will assess exercise tolerance in COPD patients. Patients will be asked to walk as fast as possible in a flat corridor, with the distance traveled in 6 min recorded. Results will be expressed in meters, with a reference value of 400–500 m for normal subjects.
5. COPD Assessment Test (CAT): The CAT [28] will assess the impact of COPD on the patient’s quality of life and track changes over time. The CAT consists of 8 questions related to cough, sputum, chest tightness, sleep, energy, emotions, and activity level. Patients will rate each item on a scale of 0–5, with the total score ranging from 0 to 40.

COPD mortality will be observed and calculated at week 52 of both the treatment and follow-up periods. Other outcome measures will be assessed every 13 weeks during the treatment period and every 26 weeks during the follow-up period.

### Sample size

Sample size estimation was based on the frequency of AEs. According to research from the Eleventh Five-Year Plan Science and Technology Support, the frequency of acute exacerbations per year for COPD patients with lung function grade 3 was 0.77 times/year in the Chinese medicine treatment group and 1.30 times/year in the standard Western medicine treatment group, with a difference of 0.53 times/year between the two groups. Sample estimation was calculated according to the sample size estimation formula for the comparison of the mean (equal number of cases) of two samples: $n = 2\left[ {\frac{{(\mu_{\alpha } + \mu_{\beta } )\sigma }}{\delta }} \right]^{2}$. Setting *α* = 0.05 (two-sided test) and *β* = 0.10, and assuming a reduction of 0.53 acute exacerbations per year in the TCM group compared to the Western medicine group, the sample size for each group was estimated to be *n* = 117, based on a bilateral cut-off value of *δ* = 0.53 and an estimated sample variance of 1.25. Considering a 20% dropout rate, the required sample size per group was adjusted to 141, with a total sample size of 282 cases for both lung function grade 3 and grade 4 patients.

Lung function grade (3–4) was used as a stratification factor for this study. Experts agreed that the clinical effect of TCM treatment would be more significant in patients with lung function grade 4, and the required sample size for these patients would be smaller than for those with grade 3. Based on this, the total sample size for the study was calculated to be 564 participants (282 × 2).

### Randomization and blinding

Patient allocation was performed using a centralized stratified block randomization method, with a block size of 4. The random sequence was generated by a computer, and the random numbers were hidden in sealed envelopes, which were kept until the end of the experiment by persons not involved in the study. Patients were randomly allocated (1:1) to the experimental or control groups based on the computer-generated random sequence. The randomization process was managed by a third-party system, which also handled data management in accordance with the study protocol. This was a double-blind trial, and all investigators and participants were blinded until the end of the trial, unless a serious adverse event or an emergency unmasking event occurred, which required emergency blinding.

### Statistical analysis

Statistical analysis was completed by SPSS V.26 (IBM). The results of the study was analyzed using the per-protocol data set (PP). The significance level was set to at a two-sided 0.05. Continuous variables were described as mean and standard deviation (*x* ± *s*), while categorical data were presented as frequency and percentage. The partially missing data of the clinical evaluation was carried forward with the principle of the last visit carried forward (LOCF). Between-group differences were compared using the independent samples *t* test or the Mann–Whitney *U*-test. Differences within a group before and after treatment were compared using the paired samples *t* test or signed rank sum test. ANOVA was used to compare repeated measures of continuous data. Statistical analyses was also conducted for the full analysis sets (FAS).

### Quality control

To ensure the reliability and integrity of the study, the strict quality control measures were implemented, including standardized procedures across all centers, centralized stratified block randomization, and double-blinding of both investigators and participants. A third-party system performed regular data monitoring and audits to ensure protocol adherence, while adverse events were systematically documented and reviewed. Data integrity was maintained by independent clinical statistician blinded to group allocation and uninvolved in providing intervention or management, and the study was overseen by an independent ethics committee to ensure compliance with ethical guidelines and participant safety.

## Results

**Fig. 1:**
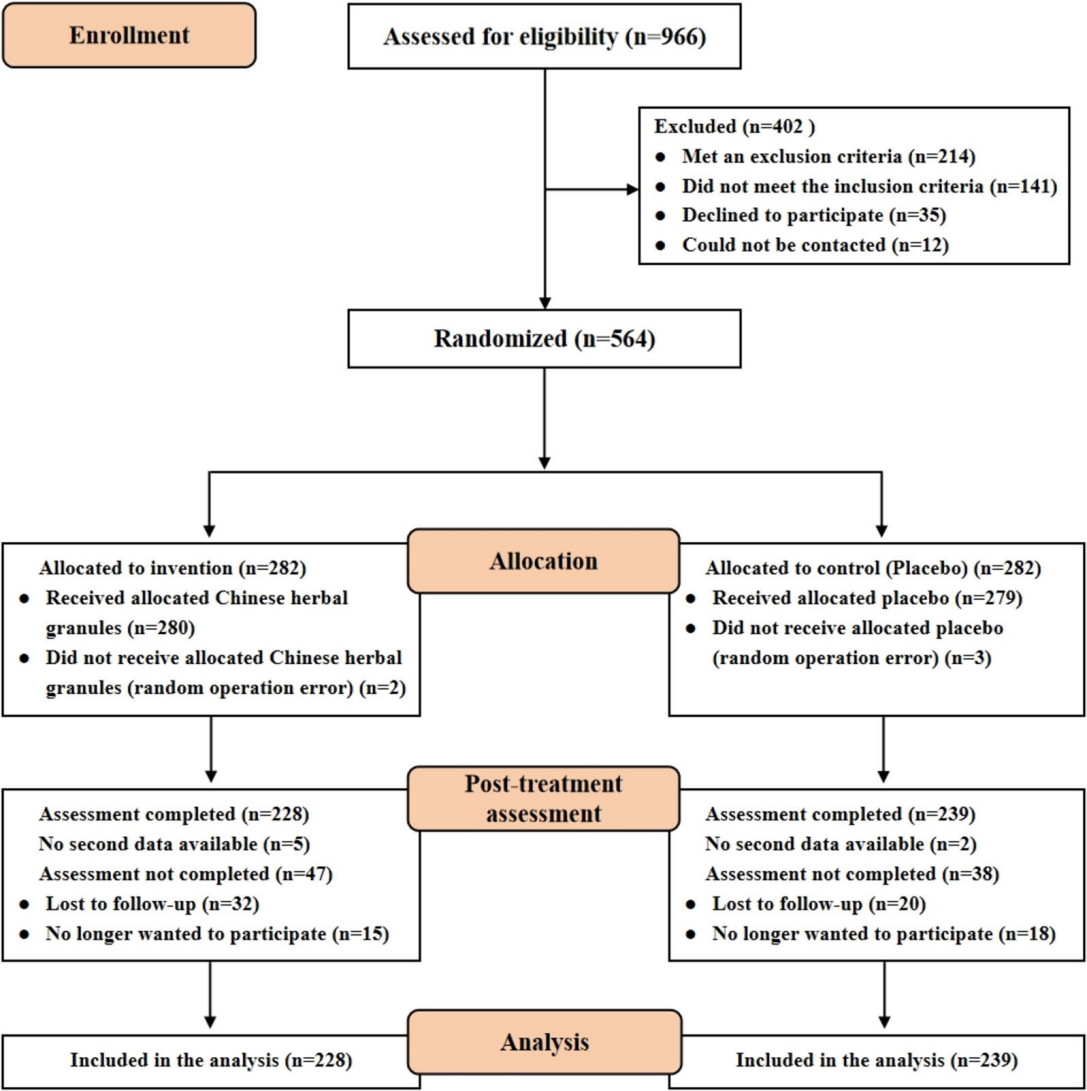
Enrollment of the patients and completion of the study

A total of 564 participants were enrolled, with 282 in each group. Twelve participants were excluded after randomization: 5 due to randomization errors at the start of the trial and 7 due to the absence of data from the second visit. During the study, 85 participants withdrew (47 from the experimental group and 38 from the control group). Ultimately, data from 467 patients (228 in the experimental group and 239 in the control group) were analyzed. The trial flowchart is presented in Fig. 1.

### Baseline clinical characteristics

There were no significant differences between the groups in terms of age, gender, lifestyle habits, body mass index (BMI), smoking status, COPD severity, disease duration, number of AEs in the previous year, lung function, mMRC, 6MWT, and CAT total score (*P* > 0.05), These results indicate comparability between the two groups, as shown in Table 2.

**Table 2:** Baseline characteristics of included COPD patients

Characteristics	Experimental group (*n* = 275)	Control group (*n* = 277)	*P* value
Age (years), mean ± SD	65.77 ± 7.79	66.18 ± 7.85	0.608
Sex, *n* (%)			0.352
Male	222 (80.7)	232 (83.8)	
Female	53 (19.3)	45 (16.2)	
BMI, mean ± SD	22.55 ± 3.19	22.52 ± 3.41	0.937
Smoking, *n* (%)			0.257
Yes	201 (73.1)	214 (77.3)	
No	74 (26.9)	63 (22.7)	
Severity of COPD, *n* (%)			0.111
GOLD 3	211 (76.7)	196 (70.8)	
GOLD 4	64 (23.3)	81 (29.2)	
Course of disease (months), mean ± SD	121.41 ± 120.91	117.84 ± 117.09	0.704
TCM syndromes, *n* (%)			0.872
Lung-spleen qi deficiency	85 (30.9)	91 (32.9)	
Lung-kidney qi deficiency	105 (38.2)	101 (36.5)	
Lung-kidney qi and yin deficiency	85 (30.9)	85 (30.7)	
Numbers of acute exacerbation, previous 12 months, mean ± SD	1.44 ± 1.17	1.42 ± 1.33	0.543
Lung function			
FVC (l), mean ± SD	2.11 ± 0.58	2.11 ± 0.64	0.776
FEV_1_ (l), mean ± SD	1.01 ± 0.55	0.95 ± 0.29	0.199
FEV_1_ (% predicted), mean ± SD	34.46 ± 8.30	35.65 ± 8.89	0.297
mMRC	2.01 ± 0.75	2.00 ± 0.81	0.443
6MWT (m), mean ± SD	330.61 ± 114.05	333.46 ± 115.07	0.771
CAT total score, mean ± SD	17.64 ± 7.38	17.91 ± 7.51	0.746
Concomitant medication			0.208
Seretide	18	16	
Spiriva	64	66	
Symbicort	56	66	
Spiriva + Symbicort	9	16	
Spiriva + Seretide	6	17	

Data analysis have been performed based on FAS. And there were no statistical differences between the two groups in any characteristic at baseline

*COPD* chronic obstructive pulmonary disease, *BMI* body mass index, *GOLD* Global Initiative for Chronic Obstructive Lung Disease, *FVC* forced vital capacity, *FEV*_*1*_ forced expiratory volume in first second, *mMRC* modified Medical Research Council dyspnea scale, *6MWT* 6 min walking test, *CAT* COPD Assessment Test, *SD* standard deviation

### Primary outcomes

#### Acute exacerbation of COPD

During the 52-week treatment period, 79 participants (34.6%) in the trial group and 112 participants (46.9%) in the control group experienced at least one AE of COPD (between-group comparison: *χ*^2^ = 10.511, *P* = 0.015). The frequency of AEs was significantly lower in the trial group compared to the control group (0.63 vs. 1.03 events per patient per year, *P* = 0.002). However, the duration of hospitalizations due to AEs did not differ significantly between the groups (Table 3). Similarly, the results from the FAS demonstrated comparable efficacy (Table S4).

During the 52-week follow-up, no significant difference was observed between the groups in the incidence of fewer than three AEs. However, 9 participants (3.9%) in the trial group and 62 participants (25.9%) in the control group experienced three or more AEs of COPD (between-group comparison: *χ*^2^ = 95.673, *P* < 0.001). The frequency of AEs in the trial group remained lower than in the control group (0.72 vs. 1.81 events per patient per year, *P* < 0.001). No significant difference in the length of hospital stays due to AEs was found between the two groups. (Table 3).

Repeated measures analysis revealed a time effect (*F* = 36.009, *P* < 0.001), a group effect (*F* = 37.759, *P* < 0.001), and a significant interaction between time and group (*F* = 29.298, *P* < 0.001) for the frequency of AEs.

**Table 3:** Comparison of the acute exacerbation

	Experimental group	Control group	*P* value*
*Treatment for 52 weeks*			
Number of AECOPD events per patient, *n* (%)			0.015
0	149 (65.4)	127 (53.1)	
1	44 (19.3)	48 (20.1)	
2	18 (7.9)	37 (15.5)	
≥3	17 (7.5)	27 (11.3)	
Number of AECOPD events per patient, mean ± SD	0.63 ± 1.15	1.03 ± 1.59	0.002
Duration (days)			
Average duration, mean ± SD	8.58 ± 6.02	8.52 ± 5.47	0.943
*Follow up for 52 weeks*			
Number of AECOPD events per patient, *n* (%)			<0.001
0	106 (46.5)	40 (16.7)	
1	86 (37.7)	62 (25.9)	
2	27 (11.8)	75 (31.4)	
≥3	9 (3.9)	62 (25.9)	
Number of AECOPD events per patient, mean ± SD	0.72 ± 0.80	1.81 ± 1.31	<0.001
Duration (days)			
Average duration, mean ± SD	8.85 ± 3.45	9.40 ± 4.91	0.981
*Two-year average number of AECOPD events*	0.68 ± 0.72	1.42 ± 1.17	<0.001
*Week 52-first visit* MD (95% CI)	−0.81 (−1.01 to −0.61)	−0.39 (−0.61 to −0.17)	
	*P* < 0.001^▲^	*P* < 0.001^▲^	
*Week 104-first visit* MD (95% CI)	−0.72 (−0.91 to −0.53)	0.39 (0.18–0.59)	
	*P* < 0.001^▲^	*P* < 0.001^▲^	

*AECOPD* acute exacerbation of chronic obstructive pulmonary disease, *MD* mean difference, *SD* standard deviation, *CI* confidence interval

* *P* values are reported for between-group comparisons

^▲^ *P* values are reported for within-group comparisons

### Secondary outcomes

#### Mortality

At the treatment and follow-up periods, six deaths occurred in each group. No statistically significant difference in mortality was observed between the two groups.

#### Lung function

**Fig. 2:**
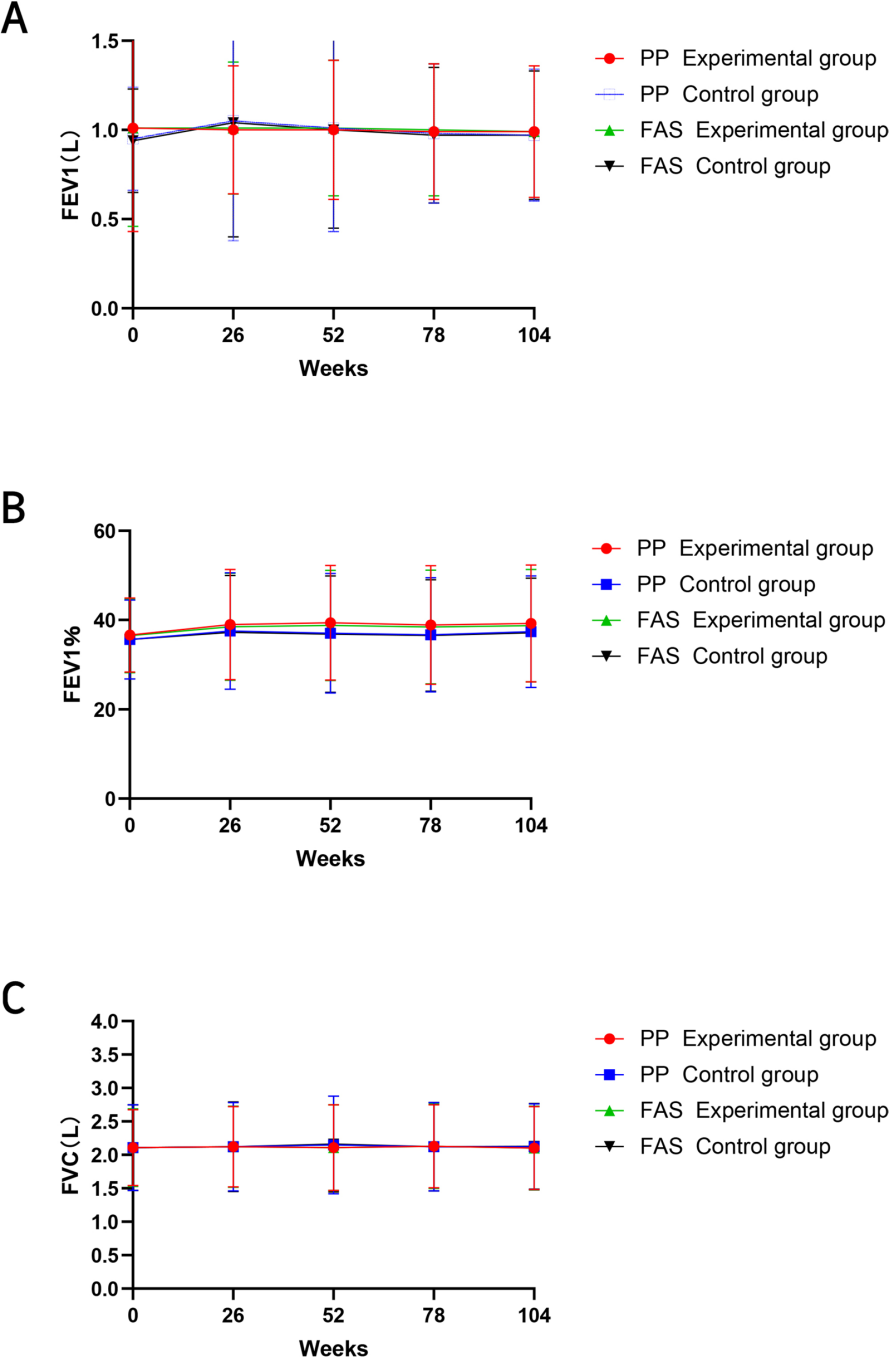
Comparison of differences in pulmonary function between groups. **A**–**C** represent intergroup differences in FEV1, FVC, and FEV1%, respectively. Lower values reflect worse pulmonary function

FEV_1_ (liters) and FVC (liters) did not differ significantly between the two groups. By the end of the 52-week treatment period, the trial group exhibited a significantly lower FEV_1_ (as a percentage) compared to the control group (mean difference [MD] −2.34, 95% CI −4.73 to −0.05, *P* = 0.019) (Table 4; Fig. 2). Similar results were observed in the FAS (Table S5). Additionally, at 26 and 52 weeks, participants in the trial group showed a slight improvement in FEV_1_ (as a percentage) (at 26 weeks: MD = 2.35, 95% CI 1.10–3.61, *P* = 0.011; at 52 weeks: MD = 2.75, 95% CI 1.25–4.24, *P* = 0.049), whereas no improvement was observed in the control group (Table S7). However, the improvement in FEV_1_% in the trial group was not sustained during the follow-up period. In the FAS, similar improvements in FEV_1_ (as a percentage) were observed at 26 and 104 weeks, with statistically significant differences at both time points (Table S8). Repeated measures analysis showed a significant between-group difference at various time points, with a time effect (*F* = 8.639, *P* < 0.001) for FEV_1_ (as a percentage).

**Table 4:** Comparison of the lung function

	Weeks 0	Week 26	Weeks 52	Weeks 78	Weeks 104
*FEV1 (l)*					
Experimental group	1.01 ± 0.58	1.01 ± 0.36	1.00 ± 0.39	0.99 ± 0.38	0.99 ± 0.37
Control group	0.95 ± 0.29	1.05 ± 0.67	1.01 ± 0.58	0.98 ± 0.39	0.97 ± 0.37
MD (95% CI)	0.06 (−0.02 to 0.14)	−0.04 (−0.14 to 0.05)	−0.01 (−0.10 to 0.08)	0.01 (−0.06 to 0.08)	0.02 (−0.05 to 0.08)
*P* value	0.414	0.758	0.503	0.524	0.710
*FEV1 (% predicted)*					
Experimental group	36.64 ± 8.32	38.99 ± 12.38	39.39 ± 12.85	38.88 ± 13.33	39.24 ± 13.13
Control group	35.65 ± 8.85	37.53 ± 13.04	37.05 ± 13.43	36.66 ± 12.83	37.36 ± 12.49
MD (95% CI)	0.99 (−0.57 to 2.55)	1.46 (−0.84 to 3.79)	2.34 (−0.05 to 4.73)	2.22 (−0.16 to 4.60)	1.88 (−4.44 to 4.21)
*P* value	0.237	0.114	0.019	0.066	0.158
*FVC (l)*					
Experimental group	2.11 ± 0.57	2.12 ± 0.60	2.11 ± 0.64	2.13 ± 0.62	2.10 ± 0.62
Control group	2.11 ± 0.64	2.12 ± 0.66	2.15 ± 0.73	2.12 ± 0.66	2.12 ± 0.64
MD (95% CI)	0.00 (−0.11 to 0.11)	0.00 (−0.11 to 0.12)	−0.04 (−0.17 to 0.09)	0.01 (−0.11 to 0.13)	−0.02 (−0.14 to 0.09)
*P* value	0.731	0.960	0.907	0.568	0.717

*MD* mean difference, *CI* confidence interval, *FEV1* forced expiratory volume in first second, *FVC* forced vital capacity

Data are mean (standard deviation). *P* values are reported for between-group comparisons

#### mMRC

**Fig. 3:**
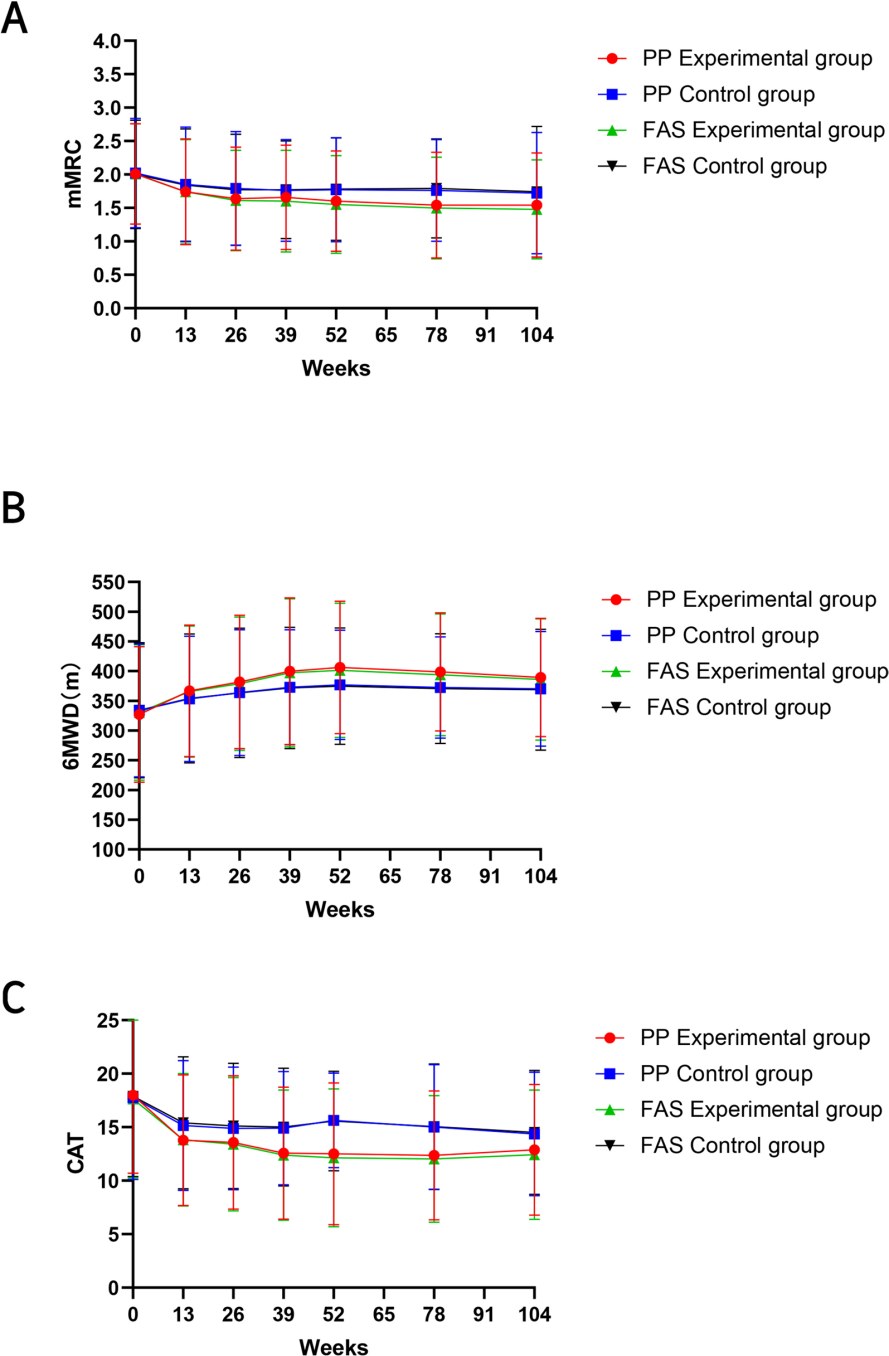
Comparison of differences between groups regarding mMRC, 6MWT, and CAT (**A**–**C**)

At weeks 52, 78, and 104, the trial group demonstrated markedly greater improvements in dyspnea compared to the control group (MD −0.17, 95% CI −0.30 to −0.03, *P* = 0.015; MD −0.22, 95% CI −0.35 to −0.075, *P* = 0.002; MD −0.18, 95% CI −0.34 to −0.03, *P* = 0.011) (Table 5; Fig. 3). Similar findings were observed in the FAS at weeks 26, 39, 52, 78, and 104, with all differences reaching statistical significance (Table S6). Repeated measures analysis revealed significant group effects (*F* = 5.556, *P* = 0.019) and time effects (*F* = 27.303, *P* < 0.001) in mMRC scores, although no interaction between time and group was observed (*F* = 1.867, *P* = 0.101).

**Table 5:** Comparison of the mMRC, 6MWT, and CAT

	Weeks 0	Week 13	Weeks 26	Weeks 39	Weeks 52	Weeks 78	Weeks 104
*mMRC (points)*							
Experimental group	2.01 ± 0.75	1.74 ± 0.79	1.64 ± 0.77	1.66 ± 0.78	1.60 ± 0.75	1.54 ± 0.79	1.54 ± 0.78
Control group	2.02 ± 0.82	1.85 ± 0.86	1.79 ± 0.85	1.76 ± 0.76	1.77 ± 0.78	1.76 ± 0.76	1.72 ± 0.91
MD (95% CI)	−0.01 (−0.15 to 0.14)	−0.11 (−0.27 to 0.03)	−0.15 (−0.30 to −0.01)	−0.10 (−0.24 to 0.04)	−0.17 (−0.30 to −0.03)	−0.22 (−0.35 to −0.07)	−0.18 (−0.34 to −0.03)
*P* value	0.999	0.130	0.057	0.129	0.015	0.002	0.011
*6MWT (m)*							
Experimental group	327.22 ± 114.33	366.88 ± 110.68	381.96 ± 112.36	399.98 ± 123.62	406.19 ± 111.52	398.78 ± 99.53	389.39 ± 99.61
Control group	333.97 ± 112.08	353.47 ± 105.48	363.76 ± 105.65	372.88 ± 96.68	376.95 ± 91.71	372.51 ± 85.10	370.16 ± 96.31
MD (95% CI)	−6.75 (−27.34 to 13.84)	13.41 (−6.24 to 33.07)	18.20 (−1.62 to 38.03)	27.10 (6.97–47.23)	29.24 (10.71–47.77)	26.27 (9.45–43.08)	19.23 (1.42–37.05)
*P* value	0.520	0.181	0.072	0.016	0.002	0.002	0.034
*CAT total score (points)*							
Experimental group	17.99 ± 7.30	13.79 ± 6.11	13.58 ± 6.23	12.57 ± 6.17	12.52 ± 6.63	12.36 ± 6.03	12.88 ± 6.11
Control group	17.76 ± 7.64	15.15 ± 6.07	14.89 ± 5.74	14.90 ± 5.29	15.63 ± 4.43	15.00 ± 5.84	14.36 ± 5.78
MD (95% CI)	0.23 (−1.13 to 1.59)	−1.36 (−2.47 to −0.25)	−1.31 (−2.40 to −0.22)	−2.33 (−3.37 to −1.29)	−3.11 (−4.13 to −2.09)	−2.64 (−3.73 to −1.57)	−1.48 (−2.57 to −0.41)
*P* value	0.633	0.040	0.017	<0.001	<0.001	<0.001	0.002

*mMRC* modified Medical Research Council dyspnea scale, *6MWT* 6-min walking test, *CAT* COPD Assessment Test, *MD* mean difference, *CI* confidence interval

Data are mean (standard deviation). *P* values are reported for between-group comparisons

In the trial group, mMRC scores were significantly reduced after 1 year of treatment (52 weeks) compared to baseline (MD −0.41, 95% CI −0.52 to −0.30, *P* < 0.001). At the 1-year follow-up (108 weeks), further reductions were observed, reaching the greatest improvement (MD −0.47, 95% CI −0.59 to −0.36, *P* < 0.001). In contrast, the control group exhibited smaller reductions in mMRC scores at both weeks 52 and 108 (MD −0.25, 95% CI −0.36 to −0.15, *P* < 0.001; MD −0.30, 95% CI −0.42 to −0.17, *P* < 0.001) (Tables S9, S10).

#### 6MWT

At weeks 39, 52, 78, and 104, the trial group demonstrated significantly greater improvements in exercise capacity compared to the control group (MD 27.10 m, 95% CI 6.97–47.23, *P* = 0.016; MD 29.24 m, 95% CI 10.71–47.77, *P* = 0.002; MD 26.27 m, 95% CI 9.45–43.08, *P* = 0.002; MD 19.23 m, 95% CI 1.42–37.05, *P* = 0.034) (Table 5; Fig. 3). Similarly, the FAS also showed statistically significant differences at weeks 39, 52, 78, and 104 (Table S6). Repeated measures analysis revealed a significant time effect (*F* = 77.833, *P* < 0.001), a significant group effect (*F* = 4.389, *P* = 0.037), and a significant interaction between time and group (*F* = 6.597, *P* = 0.014) for the 6-min walking distance.

The trial group exhibited a significant increase in 6MWT distance after 1 year of treatment (52 weeks) compared to baseline (MD 78.97 m, 95% CI 66.42–91.52, *P* < 0.001), and this improvement was maintained at the 1-year follow-up (108 weeks) (MD 62.17 m, 95% CI 49.56–74.79, *P* < 0.001). In contrast, the control group showed smaller improvements in exercise capacity at both weeks 52 and 108 (MD 49.28 m, 95% CI 32.96–53.00, *P* < 0.001; MD 36.19 m, 95% CI 24.43–47.96, *P* < 0.001) (Tables S9, S10).

#### CAT

At weeks 13, 26, 39, 52, 78, and 104, the trial group exhibited significantly reduced CAT scores compared to the control group (MD −1.36, 95% CI −2.27 to −0.25, *P* = 0.040; MD −1.31, 95% CI −2.40 to −0.22, *P* = 0.017; MD −2.33, 95% CI −3.37 to −1.29, *P* < 0.001; MD −3.11, 95% CI −4.13 to −2.09, *P* < 0.001; MD −2.64, 95% CI −3.73 to −1.57, *P* < 0.001; MD −1.48, 95% CI −2.57 to −0.41, *P* = 0.002) (Table 5; Fig. 3). Repeated measures analysis revealed significant time (*F* = 52.281, *P* < 0.001) and group effects (*F* = 16.983, *P* < 0.001), with a significant interaction between time and group (*F* = 7.013, *P* < 0.001) for CAT scores.

The trial group demonstrated a significant decrease in CAT scores after 1 year of treatment (52 weeks) compared to baseline (MD −5.47, 95% CI −6.55 to −4.40, *P* < 0.001). This improvement was sustained at the 1-year follow-up (108 weeks) (MD −5.11, 95% CI −6.26 to −3.97, *P* < 0.001). In contrast, the control group exhibited smaller reductions in CAT scores at both week 52 and week 108 (MD −2.13, 95% CI −3.08 to −1.18, *P* < 0.001; MD −3.40, 95% CI −4.53 to −2.26, *P* < 0.001). (Tables S9, S10).

#### Adverse events

Throughout the treatment and follow-up phases, all participants exhibited normal biochemical profiles, urinalysis, and electrocardiograms. A total of 112 adverse events were recorded by 78 participants. The most common adverse event in both groups was acute upper respiratory infection. Fifteen serious adverse events were recorded, including three cases of malignant tumors and 12 deaths. In the experimental group, six deaths were reported: one due to acute myocardial infarction, one from malignant arrhythmia, and four following severe AEs of COPD. In the control group, six deaths occurred: one due to acute myocardial infarction and five due to severe AEs of COPD. None of the 15 serious adverse events were deemed related to the intervention (Table 6).

**Table 6:** Adverse events in the experimental and control group

Type of adverse event	Experimental group	Control group
Acute upper respiratory infections	39	45
Death	6^a^	6^a^
Hypertension	3	4
Acute gastroenteritis	2	3
Type 2 diabetes	1	2
Lung cancer	1^a^	1^a^
Coronary heart disease	1	1
Benign prostatic hyperplasia	0	2
Gastric cancer	1^a^	0
Hemorrhoids	1	0
Kidney stone	0	1
Gall stone	1	0
Urinary tract infection	1	0

^a^Serious adverse events, that were not considered to be related to the treatment

## Discussion

TCM treatment in this study involved three types of herbal granules: Bu-Fei Jian-Pi Granules, Bu-Fei Yi-Shen Granules, and Yi-Qi Zi-Shen Granules. These granules are produced based on traditional Chinese formulas and are composed of carefully selected, highly concentrated herbs. Each type of granule corresponds to a specific TCM syndrome, with the choice of treatment based on the patient’s individual pattern, determined through TCM diagnostic principles related to the lung, spleen, and kidney. This comprehensive therapy based on TCM patterns aligns with the current multidimensional disease typing in Western medicine, which aims to enhance personalized patient care. Various typing methods are being explored, including lung function trajectory typing, imaging assessment of disease phenotypes, and typing based on gene expression profiles. Our study has observed a therapeutic advantage of TCM treatment in patients with severe to very severe COPD. Compared to the control group, the frequency of AEs in the trial group showed a reduction during the 52-week treatment and the subsequent 52-week follow-up period.

However, the duration of AEs between the two groups was not statistically significant. Moreover, improvements were noted in dyspnea, exercise capacity, and CAT scores, although lung function showed minimal to no improvement in severe to very severe COPD patients post-treatment. To our knowledge, this is the largest, multi-center, randomized controlled trial employing TCM treatment with syndrome differentiation for severe to very severe COPD patients. Our findings underscore a patient-centered approach and an emphasis on individualized treatment, providing valuable insights for clinical practice, especially for patients with severe and very severe COPD.

AEs are a primary outcome in this study, significantly impacting the decline in lung function and health-related quality of life in COPD patients [29]. AEs also lead to hospitalizations and poorer prognosis, particularly in severe to very severe COPD. It has been reported that the mortality rate for patients admitted to the intensive care unit due to AEs is approximately 11–24% [30], and among COPD patients hospitalized for AEs, severe to very severe patients account for 71.1% [31]. The incidence rate of AEs within 1 year for COPD patients in China is as high as 43.7% [32]. Therefore, reducing the frequency and duration of AEs is a key therapeutic goal, especially for severe to very severe COPD patients. In this study, compared to the control group, the trial group experienced a reduction in AEs after 1 year of treatment, with statistically significant differences compared to the control group. A previous study found that YuPingFeng granules were associated with a significantly lower rate of AEs compared to placebo (1.15 vs. 1.55 events, per patient-year) [33]. Another study reported that JianPiYiFei II granules had a significantly lower rate of AEs compared to placebo (0.87 vs. 1.34 events, per patient-year) [34]. Our study showed a similar reduction in AEs (0.63 events in the trial group vs. 1.03 events in the control group). However, neither of these prior studies included follow-up observations post-treatment, leaving the long-term efficacy unverified. In contrast, our study evaluated long-term efficacy with a 1-year follow-up after treatment cessation (0.72 vs. 1.81 events), thus confirming the sustained benefits of Chinese herbal granules. These results warrant further dissemination.

As lung function worsens, the exercise capacity of COPD patients also deteriorates, particularly in those with severe to very severe COPD. The 6MWT is a key measure of exercise capacity in COPD patients and correlates with clinical outcomes such as hospitalization and mortality rates [35]. While modern medical interventions primarily focus on the lungs, often neglecting peripheral tissues, non-pharmacological treatments like pulmonary rehabilitation have shown some improvements, though the evidence remains suboptimal [36]. The 6MWT serves as an essential tool for determining patient eligibility for rehabilitation programs, with a threshold of approximately 350 m and a minimal clinically important difference of 25 m [37, 38]. After 52 weeks of treatment, the Chinese herbal granules demonstrated a longer 6-min walking distance compared to the placebo (29.24 m). Following a 52-week follow-up, the Chinese herbal granules exhibited an additional 19.23 m compared to the placebo group, suggesting that Chinese herbal granules may enhance pulmonary ventilation, reduce hypoxia, and thereby improve muscle strength and exercise endurance.

Dyspnea is a common clinical symptom in COPD patients, often exacerbated as lung function deteriorates, affecting quality of life and prognosis [39]. Despite standard treatment, 43% of COPD patients continue to experience persistent dyspnea, with an mMRC score of 2 or higher [39]. TCM, grounded in the observation of symptoms and physical signs, demonstrated its efficacy through symptom improvement. In this study, compared to the placebo group, the TCM treatment resulted in significant reductions in mMRC scores in severe to very severe COPD patients, with more pronounced improvements from week 13 onward. These results suggest the long-term effects of TCM, which are often overlooked in clinical research. Additionally, the CAT score is a globally recognized scale that gauges the impact of COPD on health and quality of life, correlating positively with the frequency of acute exacerbations in the preceding year [40]. Our study revealed that TCM granules can reduce CAT scores, thus alleviating disease severity in severe to very severe COPD patients.

Previous studies have shown that the Bufei formula, Bufei Jianpi formula, and Bufei Yishen formula can play therapeutic roles in COPD through multiple molecular mechanisms. For instance, the Bufei Yishen formula can regulate the Th17/Treg cell balance by activating the adenosine receptor 2a [41, 42], inhibit the mTOR signaling pathway to suppress macrophage M2 polarization and improve airway remodeling in COPD rats [43], inhibit the MAPK signaling pathway to reduce macrophage inflammatory responses [44], activate Nrf2 to suppress oxidative stress and ameliorate COPD-induced airway epithelial cell aging [45], and inhibit the EGFR signaling pathway to protect the airway epithelial cell barrier [46]. The Bufei Jianpi formula can enhance mucosal immunity by regulating the SCFAs/GPR43/NLRP3 axis to restore intestinal flora, and protect COPD skeletal muscle by modulating the AMPK signaling pathway to improve mitochondrial function [47, 48]. The Bufei formula can inhibit inflammation through pathways involving Akt1, BCL2, and NF-κB [49]. These findings indicate that the Bufei formula, Bufei Jianpi formula, and Bufei Yishen formula can ameliorate COPD through various mechanisms, such as inhibiting inflammation, oxidative stress, and aging. These studies provide a theoretical basis for the clinical application of traditional Chinese medicine formulas in the treatment of COPD.

Chronic treatment and polypharmacy may lead to adverse events, especially considering that most COPD patients are elderly and with multiple comorbidities. Thus, safety assessment is of utmost importance. The trial showed that the type and frequency of adverse events were comparable between the treatment and placebo groups, with most being mild, indicating that long-term oral use of Chinese herbal granules is safe and well-tolerated.

However, this study has several limitations. First, the herbal interventions (granules) of the three TCM patterns in this study were considered as comprehensive interventions rather than individual herbal treatments for each pattern. The study aimed to evaluate the comprehensive interventions of the three common patterns for patients with severe and very severe COPD. Therefore, the effectiveness of each herbal intervention on the TCM patterns was not assessed. Second, the total sample size was calculated for the overall study, which could result in heterogeneous outcomes across different subgroups. Furthermore, the dropout rate was higher than expected, resulting in the inability to perform subgroup analysis.

## Conclusion

In conclusion, the comprehensive therapy based on TCM patterns demonstrated both safety and efficacy in treating severe and very severe COPD patients. This approach effectively reduced the frequency of AEs, alleviated dyspnea, enhanced exercise tolerance, and improved clinical symptoms. However, no significant improvement in lung function was observed. Further research is necessary to investigate the potential effects of this treatment on lung function in COPD patients.

## Supplementary Information

**Additional file 1.**

## Data Availability

The datasets used in this study are available from the corresponding author on reasonable request.

## Abbreviations

TCM: Traditional Chinese medicine
COPD: Chronic obstructive pulmonary disease
AEs: Acute exacerbations
6MWT: 6-Minute walking test
CAT: COPD assessment test
ICS: Inhaled corticosteroids
LABA: Long-acting β2-agonists
LAMA: Long-acting muscarinic antagonists
GOLD: Global Initiative for Chronic Obstructive Lung Disease
FVC: Forced vital capacity
FEV_1_: Forced expiratory volume in one second
FEV_1_%: Forced expiratory volume in one second percentage of the predicted value
mMRC: Modified Medical Research Council
BMI: Body mass index
