# Supplementary material for: Effect of traditional Chinese medicine combined with conventional Western medicine for patients with severe/very severe chronic obstructive pulmonary disease: a multi-center, randomized, double-blind, controlled study
Source: Chin Med. 2025 May 20;20:66. doi: 10.1186/s13020-025-01117-x (PMC12093656; doi:10.1186/s13020-025-01117-x)
Supplement: Supplementary file 1 — Additional file 1. [file 13020_2025_1117_MOESM1_ESM.docx]

**Supplementary Materials**

**Table S1 Study participating hospitals**

| Number | Hospital |
| --- | --- |
| 1 | The First Affiliated Hospital of Henan University of Chinese Medicine |
| 2 | The Second Affiliated Hospital of Tianjin University of Traditional Chinese Medicine |
| 3 | First Teaching Hospital of Tianjin University of Traditional Chinese Medicine |
| 4 | The Shuguang Hospital affiliated to Shanghai University of Traditional Chinese Medicine |
| 5 | The First Affiliated Hospital of Anhui University of Chinese Medicine |
| 6 | The First Affiliated Hospital of Guangzhou University of Chinese Medicine |
| 7 | Guangdong Province Hospital of Traditional Chinese Medicine |
| 8 | the Affiliated Hospital of Jiangxi University of Chinese Medicine |
| 9 | Jiangsu Province Hospital of Chinese Medicine |
| 10 | Liaoning Province Chinese Medicine Institute |

**Table S2 Diagnostic criteria in TCM**

| TCM syndrome | Symptoms | Diagnostic criteria |
| --- | --- | --- |
| Lung-spleen qi deficiency | (1) cough, wheezing, shortness of breath, and worsening with movement;  (2) fatigue, self sweating, and worsening with movement;  (3) easy to catch a cold,  (4) poor appetite or insufficient food intake;  (5) abdominal distension, bloating, or loose stools;  (6) tongue fat or with teeth marks, pale tongue, thin white or greasy moss, dull or slow or weak pulse. | Presence of any two of (1), (2) or (3) with any two of (4), (5) or (6). |
| Lung-kidney qi deficiency | (1) wheezing, shortness of breath, aggravated by movement;  (2) weakness, spontaneous sweating, aggravated by movement;  (3) easy to catch a cold;  (4) soreness and weakness of the waist and knees;  (5) ringing in the ears, dizziness, or facial weakness and floatation;  (6) frequency of urination, increase in nocturia, or loss of urine in coughing and wheezing;  (7) pale tongue and white coating, and a hollow, thin, or weak pulse. | Presence of any two of (1), (2) or (3), together with any of (4), (5), (6) or (7). |
| Lung-kidney qi and yin deficiency | (1) wheezing, shortness of breath, aggravated by movement;  (2) sweating or fatigue, aggravated by movement;  (3) easy to catch a cold;  (4) waist and knee soreness and weakness;  (5) ringing in the ears, or dizziness or dizziness;  (6) dry cough or sputum, sputum is not refreshing;  (7) night sweating;  (8) hands and feet, heart fever;  (9) pale or red tongue, thin or flaky tongue coating, or sunken or weak or fine pulse. | Presence of any two of (1), (2) or (3), any of (4) or (5), and any of (6), (7), (8) or (9). |

**Table S3 Main components of TCM placebos**

| **Ingredient name** | **Ingredient ratio (%)** |
| --- | --- |
| **Bu-Fei Jian-Pi placebos** | |
| Lactose | 81.743 |
| Maltodextrin | 17.044 |
| Lemon yellow colouring (edible) | 0.166 |
| Sunset yellow colouring (edible) | 0.017 |
| Caramel colouring (edible) | 1.030 |
| Sucralose | 0.010 |
| **Bu-Fei Yi-Shen placebos** | |
| Lactose | 76.134 |
| Maltodextrin | 22.234 |
| Lemon yellow colouring (edible) | 0.183 |
| Sunset yellow colouring (edible) | 0.027 |
| Caramel colouring (edible) | 1.420 |
| Bitter acid | 0.002 |
| **Yi-Qi Zi-Shen placebos** | |
| Lactose | 77.586 |
| Maltodextrin | 20.067 |
| Lemon yellow colouring (edible) | 0.133 |
| Sunset yellow colouring (edible) | 0.012 |
| Caramel colouring (edible) | 2.200 |
| Bitter acid | 0.002 |

**Table S4 Comparison of the acute exacerbation (Full analysis set)**

|  | **Experimental group** | **Control group** | ***P* value**^✱^ |
| --- | --- | --- | --- |
| **Treatment for 52 weeks** |  |  |  |
| Number of AECOPD events per patient, n (%) |  |  | 0.067 |
| 0 | 159（57.8） | 138（49.8） |  |
| 1 | 64（23.3） | 60（21.7） |  |
| 2 | 31（11.3） | 47（17.0） |  |
| ≥3 | 21（7.6） | 32（11.6） |  |
| Number of AECOPD events per patient, mean ± SD | 0.75±1.20 | 1.08±1.56 | 0.016 |
| Duration (days) |  |  |  |
| Average duration, mean ± SD | 8.09±5.38 | 7.94±4.91 | 0.523 |
| **Follow up for 52 weeks** |  |  |  |
| Number of AECOPD events per patient, n (%) |  |  | ＜0.001 |
| 0 | 116（42.2） | 50（18.1） |  |
| 1 | 105（38.2） | 75（27.1） |  |
| 2 | 41（14.9） | 86（31.0） |  |
| ≥3 | 13（4.7） | 66（23.8） |  |
| Number of AECOPD events per patient, mean ± SD | 0.83±0.92 | 1.75±1.31 | ＜0.001 |
| Duration(days) |  |  |  |
| Average duration, mean ± SD | 8.03±3.02 | 8.25±4.59 | 0.336 |
| **Two-year average number of AECOPD events** | 0.79±0.86 | 1.42±1.18 | ＜0.001 |
| **Week 52-first visit**  MD (95% CI) | -0.81（-0.98 to -0.63） | -0.48（-0.68 to -0.28） |  |
|  | *P*＜0.001^▲^ | *P*＜0.001^▲^ |  |
| **Week 104-first visit**  MD (95% CI) | -0.52（-0.71 to -0.35） | -0.14（-0.33 to 0.05） |  |
|  | *P*＜0.001^▲^ | *P*＜0.001^▲^ |  |

**Abbreviations:** AECOPD, acute exacerbation of chronic obstructive pulmonary disease; MD, mean difference; SD, standard deviation; CI, confidence interval.

^✱^ P values are reported for between-group comparisons. ^▲^P values are reported for within-group comparisons.

**Table S5 Comparison of the lung function. (Full analysis set)**

|  | Weeks 0 | Week 26 | Weeks 52 | Weeks 78 | Weeks 104 |
| --- | --- | --- | --- | --- | --- |
| **FEV1 (litres)** |  |  |  |  |  |
| Experimental group | 1.01±0.55 | 1.01±0.37 | 1.01±0.38 | 1.00±0.37 | 0.99±0.37 |
| Control group | 0.94±0.29 | 1.04±0.64 | 1.00±0.55 | 0.97±0.38 | 0.97±0.36 |
| MD (95% CI) | 0.07（-0.01 to 0.14） | -0.03（-0.11 to 0.06） | -0.01（-0.08 to 0.08） | 0.03（-0.04 to 0.09） | 0.02（-0.03 to 0.09） |
| *P* value | 0.199 | 0.501 | 0.274 | 0.267 | 0.404 |
| **FEV1 (% predicted)** |  |  |  |  |  |
| Experimental group | 36.46±8.30 | 38.46±12.01 | 38.79±12.37 | 38.42±12.76 | 38.72±12.59 |
| Control group | 35.65±8.89 | 37.22±12.73 | 36.82±13.08 | 36.54±12.54 | 37.14±12.25 |
| MD (95% CI) | 0.81（-0.63 to 2.25） | 1.23（-0.84 to 3.30） | 1.97（-0.16 to 4.10） | 1.88（-0.24 to 3.99） | 1.58（-0.50 to 3.66） |
| *P* value | 0.297 | 0.157 | 0.026 | 0.079 | 0.166 |
| **FVC (litres)** |  |  |  |  |  |
| Experimental group | 2.11±0.58 | 2.12±0.61 | 2.11±0.64 | 2.13±0.63 | 2.10±0.63 |
| Control group | 2.11±0.64 | 2.12±0.67 | 2.16±0.72 | 2.12±0.66 | 2.13±0.64 |
| MD (95% CI) | 0.00（-0.10 to 0.10） | 0.00（-0.11 to 0.10 ） | -0.05（-0.16 to 0.07） | 0.01（-0.10 to 0.11） | -0.03（-0.13 to 0.09） |
| *P* value | 0.776 | 0.755 | 0.746 | 0.694 | 0.773 |

**Abbreviations:** MD, mean difference; CI, confidence interval; FEV1, forced expiratory volume in first second; FVC, forced vital capacity.

Data are mean (Standard deviation). *P* values are reported for between-group comparisons.

**Table S6 Comparison of the mMRC, 6MWT, and CAT. (Full analysis set)**

|  | Weeks 0 | Week 13 | Weeks 26 | Weeks 39 | Weeks 52 | Weeks 78 | Weeks 104 |
| --- | --- | --- | --- | --- | --- | --- | --- |
| **mMRC (points)** |  |  |  |  |  |  |  |
| Experimental group | 2.01±0.75 | 1.74±0.78 | 1.61±0.75 | 1.60±0.76 | 1.55±0.73 | 1.50±0.76 | 1.48±0.74 |
| Control group | 2.00±0.81 | 1.84±0.84 | 1.77±0.83 | 1.77±0.73 | 1.78±0.77 | 1.79±0.74 | 1.74±0.98 |
| MD (95% CI) | -0.01  （-0.12 to 0.15） | -0.10  （-0.23 to 0.04） | -0.16  （-0.30 to -0.01） | -0.10  （-0.28 to -0.02） | -0.23  （-0.35 to -0.10） | -0.29  （-0.41 to -0.16） | -0.26  （-0.40 to -0.13） |
| *P* value | 0.733 | 0.172 | 0.031 | 0.003 | ＜0.001 | ＜0.001 | ＜0.001 |
| **6MWT (m)** |  |  |  |  |  |  |  |
| Experimental group | 330.52±114.03 | 365.64±110.27 | 378.94±112.37 | 397.34±124.48 | 401.18±112.82 | 393.84±102.37 | 386.11±102.03 |
| Control group | 334.17±113.61 | 354.03±108.37 | 363.59±108.90 | 371.64±102.21 | 374.74±97.83 | 370.65±92.52 | 368.62±101.52 |
| MD (95% CI) | -3.66  （-22.69 to 15.37） | 11.61  （-6.67 to 29.89） | 15.35  （-3.15 to 33.85） | 25.70  （6.66 to 44.73） | 26.44  （8.78 to 44.08） | 23.19  （6.88 to 39.50） | 17.49  （0.47 to 34.51） |
| *P* value | 0.706 | 0.213 | 0.104 | 0.008 | 0.003 | 0.005 | 0.026 |
| **CAT total score (points)** |  |  |  |  |  |  |  |
| Experimental group | 17.63±7.37 | 13.82±6.20 | 13.41±6.24 | 12.39±6.08 | 12.13±6.45 | 12.03±5.91 | 12.42±6.05 |
| Control group | 17.89±7.50 | 15.40±6.17 | 15.12±5.86 | 15.02±5.51 | 15.58±4.66 | 15.06±5.86 | 14.52±5.81 |
| MD (95% CI) | -0.26  （-1.51 to 0.98） | -1.58  （-2.61 to -0.54） | -1.71  （-2.72 to -0.69） | -2.63  （-3.60 to -1.66） | -3.45  （-4.40 to -2.51） | -3.03  （-4.02 to -2.05） | -2.10  （-3.09 to -1.11） |
| *P* value | 0.756 | 0.009 | 0.001 | ＜0.001 | ＜0.001 | ＜0.001 | ＜0.001 |

**Abbreviations:** mMRC, modified Medical Research Council dyspnea scale; 6MWT, 6-minute walking test; CAT, COPD Assessment Test; MD, mean. difference; CI, confidence interval.

Data are mean (Standard deviation). *P* values are reported for between-group comparisons.

**Table S7 Within-group comparisons of lung function. (Per-protocol analysis set)**

|  | Time Point | Group | Z/t | P-Value | MD (95% CI) |
| --- | --- | --- | --- | --- | --- |
| FEV1 (litres) | Week 26 -first visit | Experimental group | -0.764 | 0.445 | 0.00 (-0.07 to 0.08) |
|  |  | Control group | -1.172 | 0.241 | 0.10 (0.02 to 0.18) |
|  | Week 52 -first visit | Experimental group | -0.281 | 0.779 | -0.01 (-0.09 to 0.07) |
|  |  | Control group | -0.435 | 0.663 | 0.06 (-0.01 to 0.13) |
|  | Week 78 -first visit | Experimental group | -0.436 | 0.663 | -0.02 (-0.10 to 0.06) |
|  |  | Control group | -0.656 | 0.512 | 0.03 (-0.01 to 0.06) |
|  | Week 104 -first visit | Experimental group | -0.262 | 0.793 | -0.02 (-0.11 to 0.05) |
|  |  | Control group | -0.568 | 0.570 | 0.02 (-0.02 to 0.06) |
| FEV_1_ (% predicted) | Week 26 -first visit | Experimental group | -2.528 | 0.011 | 2.35 (1.10 to 3.61) |
|  |  | Control group | -1.314 | 0.189 | 1.88 (0.58 to 3.17) |
|  | Week 52 -first visit | Experimental group | -1.972 | 0.049 | 2.75 (1.25 to 4.24) |
|  |  | Control group | -0.086 | 0.931 | 1.40 (0.03 to 2.75) |
|  | Week 78 -first visit | Experimental group | -1.516 | 0.130 | 2.24 (0.71 to 3.76) |
|  |  | Control group | -0.032 | 0.974 | 1.01 (-0.28 to 2.29) |
|  | Week 104 -first visit | Experimental group | -2.613 | 0.009 | 2.60 (1.12 to 4.08) |
|  |  | Control group | -1.339 | 0.180 | 1.70 (0.45 to 2.96) |
| FVC (litres) | Week 26 -first visit | Experimental group | 0.479 | 0.632 | 0.01 (-0.05 to 0.07) |
|  |  | Control group | -0.209 | 0.834 | 0.01 (-0.05 to 0.08) |
|  | Week 52 -first visit | Experimental group | 0.202 | 0.840 | 0.00 (-0.06 to 0.08) |
|  |  | Control group | -0.785 | 0.432 | 0.04 (-0.02 to 0.12) |
|  | Week 78 -first visit | Experimental group | 0.63 | 0.530 | 0.02 (-0.05 to 0.09) |
|  |  | Control group | -0.314 | 0.753 | 0.01 (-0.05 to 0.08) |
|  | Week 104 -first visit | Experimental group | -0.142 | 0.887 | -0.01 (-0.08 to 0.06) |
|  |  | Control group | 0.535 | 0.593 | 0.01 (-0.05 to 0.08) |

**Abbreviations:** FEV1, forced expiratory volume in first second; FVC, forced vital capacity; MD, mean difference; CI, confidence interval.

**Table S8 Within-group comparisons of lung function. (Full analysis set)**

|  | Time Point | Group | Z/t | P-Value | MD (95% CI) |
| --- | --- | --- | --- | --- | --- |
| FEV1 (litres) | Week 26 -first visit | Experimental group | -0.793 | 0.428 | 0.00 (-0.07 to 0.06) |
|  |  | Control group | -1.257 | 0.209 | 0.09 (0.02 to 0.16) |
|  | Week 52 -first visit | Experimental group | -0.352 | 0.725 | 0.01 (-0.07 to 0.06) |
|  |  | Control group | -0.297 | 0.767 | 0.06 (-0.01 to 0.11) |
|  | Week 78 -first visit | Experimental group | -0.359 | 0.719 | -0.01 (-0.08 to 0.05) |
|  |  | Control group | -0.544 | 0.586 | 0.02 (-0.01 to 0.06) |
|  | Week 104 -first visit | Experimental group | -0.180 | 0.857 | -0.02 (-0.09 to 0.05) |
|  |  | Control group | -0.452 | 0.652 | 0.02 (-0.01 to 0.05) |
| FEV_1_ (% predicted) | Week 26 -first visit | Experimental group | -2.395 | 0.017 | 1.99 (0.91 to 3.08) |
|  |  | Control group | -1.222 | 0.222 | 1.57 (0.44 to 2.71) |
|  | Week 52 -first visit | Experimental group | -1.784 | 0.074 | 2.33 (1.06 to 3.59) |
|  |  | Control group | -0.092 | 0.927 | 1.17 (-0.03 to 2.37) |
|  | Week 78 -first visit | Experimental group | -1.508 | 0.132 | 1.95 (0.66 to 3.25) |
|  |  | Control group | -1.102 | 0.270 | 0.89 (-0.24 to 2.02) |
|  | Week 104 -first visit | Experimental group | -2.552 | 0.011 | 2.25 (0.99 to 3.51) |
|  |  | Control group | -1.395 | 0.163 | 1.49 (0.38 to 2.59) |
| FVC (litres) | Week 26 -first visit | Experimental group | -0.741 | 0.458 | 0.02 (-0.04 to 0.07) |
|  |  | Control group | -0.024 | 0.981 | 0.02 (-0.04 to 0.07) |
|  | Week 52 -first visit | Experimental group | -0.432 | 0.666 | 0.00 (-0.05 to 0.06) |
|  |  | Control group | -1.029 | 0.304 | 0.05 (-0.01 to 0.11) |
|  | Week 78 -first visit | Experimental group | -0.289 | 0.772 | 0.02 (-0.04 to 0.08) |
|  |  | Control group | -0.338 | 0.736 | 0.01 (-0.04 to 0.07) |
|  | Week 104 -first visit | Experimental group | -0.760 | 0.447 | 0.00 (-0.06 to 0.06) |
|  |  | Control group | -0.017 | 0.987 | 0.02 (-0.04 to 0.07) |

**Abbreviations:** FEV1, forced expiratory volume in first second; FVC, forced vital capacity; MD, mean difference; CI, confidence interval.

**Table S9 Within-group comparisons of mMRC, 6MWD, and CAT. (Per-protocol analysis set)**

|  | Time Point | Group | Z/t | P-Value | MD (95% CI) |
| --- | --- | --- | --- | --- | --- |
| **mMRC** | Week 13 -first visit | Experimental group | -5.53 | <0.001 | -0.27 (-0.36 to -0.18) |
|  |  | Control group | -3.606 | <0.001 | -0.17 (-0.25 to -0.075) |
|  | Week 26 -first visit | Experimental group | -6.585 | <0.001 | -0.37 (-0.48 to -0.27) |
|  |  | Control group | -4.208 | <0.001 | -0.23 (-0.33 to -0.13) |
|  | Week 39 -first visit | Experimental group | -5.661 | <0.001 | -0.35 (-0.47 to -0.23) |
|  |  | Control group | -5.14 | <0.001 | -0.26 (-0.35 to -0.17) |
|  | Week 52 -first visit | Experimental group | -6.466 | <0.001 | -0.41 (-0.52 to -0.30) |
|  |  | Control group | -4.576 | <0.001 | -0.25 (-0.36 to -0.15) |
|  | Week 78 -first visit | Experimental group | -7.272 | <0.001 | -0.47 (-0.58 to -0.35) |
|  |  | Control group | -4.434 | <0.001 | -0.26 (-0.37 to -0.14) |
|  | Week 104 -first visit | Experimental group | -7.075 | <0.001 | -0.47 (-0.59 to -0.36) |
|  |  | Control group | -4.567 | <0.001 | -0.30 (-0.42 to -0.17) |
| **6MWT (m)** | Week 13 -first visit | Experimental group | 7.94 | <0.001 | 39.66 (29.82 to 49.51) |
|  |  | Control group | 4.869 | <0.001 | 19.50 (11.61 to 27.39) |
|  | Week 26 -first visit | Experimental group | 10.597 | <0.001 | 54.74 (44.56 to 64.92) |
|  |  | Control group | 6.53 | <0.001 | 29.79 (20.80 to 38.78) |
|  | Week 39 -first visit | Experimental group | 11.473 | <0.001 | 72.76 (60.26 to 85.25) |
|  |  | Control group | 7.924 | <0.001 | 38.91 (29.23 to 48.58) |
|  | Week 52 -first visit | Experimental group | 12.402 | <0.001 | 78.97 (66.42 to 91.52) |
|  |  | Control group | 8.45 | <0.001 | 42.98 (32.96 to 53.00) |
|  | Week 78 -first visit | Experimental group | 11.898 | <0.001 | 71.54 (59.71 to 83.41) |
|  |  | Control group | 7.364 | <0.001 | 38.54 (28.23 to 48.86) |
|  | Week 104 -first visit | Experimental group | 9.711 | <0.001 | 62.17 (49.56 to 74.79) |
|  |  | Control group | 6.062 | <0.001 | 36.19 (24.43 to 47.96) |
| **CAT** | Week 13 -first visit | Experimental group | -9.196 | <0.001 | -4.20 (-4.97 to -3.44) |
|  |  | Control group | -6.761 | <0.001 | -2.61 (-3.35 to -1.87) |
|  | Week 26 -first visit | Experimental group | -8.349 | <0.001 | -4.41 (-5.32 to -3.51) |
|  |  | Control group | -6.35 | <0.001 | -2.87 (-3.72 to -2.02) |
|  | Week 39 -first visit | Experimental group | -8.898 | <0.001 | -5.42 (-6.42 to -4.43) |
|  |  | Control group | -5.259 | <0.001 | -2.86 (-3.83 to -1.89) |
|  | Week 52 -first visit | Experimental group | -8.464 | <0.001 | -5.47 (-6.55 to -4.40) |
|  |  | Control group | -3.961 | <0.001 | -2.13 (-3.08 to -1.18) |
|  | Week 78 -first visit | Experimental group | -8.319 | <0.001 | -5.63 (-6.77 to -4.50) |
|  |  | Control group | -4.425 | <0.001 | -2.76 (-3.86 to -1.65) |
|  | Week 104 -first visit | Experimental group | -7.571 | <0.001 | -5.11 (-6.26 to -3.97) |
|  |  | Control group | -5.192 | <0.001 | -3.40 (-4.53 to -2.26) |

**Abbreviations:** mMRC, modified Medical Research Council dyspnea scale; 6MWT, 6-minute walking test; CAT, COPD Assessment Test; MD, mean difference; CI, confidence interval.

**Table S10 Within-group comparisons of mMRC, 6MWD, and CAT. (Full analysis set)**

|  | Time Point | Group | Z/t | P-Value | MD (95% CI) |
| --- | --- | --- | --- | --- | --- |
| **mMRC** | Week 13 -first visit | Experimental group | -6.185 | <0.001 | -0.27 (-0.35 to -0.19) |
|  |  | Control group | -3.907 | <0.001 | -0.16 (-0.24 to -0.08) |
|  | Week 26 -first visit | Experimental group | -7.384 | <0.001 | -0.40 (-0.49 to -0.30) |
|  |  | Control group | -4.752 | <0.001 | -0.24 (-0.33 to -0.14) |
|  | Week 39 -first visit | Experimental group | -7.028 | <0.001 | -0.42 (-0.52 to -0.31) |
|  |  | Control group | -4.808 | <0.001 | -0.23 (-0.32 to -0.14) |
|  | Week 52 -first visit | Experimental group | -7.758 | <0.001 | -0.47 (-0.57 to -0.36) |
|  |  | Control group | -4.352 | <0.001 | -0.22 (-0.32 to -0.13) |
|  | Week 78 -first visit | Experimental group | -8.546 | <0.001 | -0.51 (-0.62 to -0.41) |
|  |  | Control group | -3.907 | <0.001 | -0.21 (-0.32 to -0.11) |
|  | Week 104 -first visit | Experimental group | -8.488 | <0.001 | -0.54 (-0.64 to -0.43) |
|  |  | Control group | -4.301 | <0.001 | -0.26 (-0.37 to -0.14) |
| **6MWT (m)** | Week 13 -first visit | Experimental group | 8.141 | <0.001 | 35.13 (26.63 to 43.62) |
|  |  | Control group | 5.440 | <0.001 | 19.86 (12.67 to 27.05) |
|  | Week 26 -first visit | Experimental group | 10.829 | <0.001 | 48.43 (39.62 to 57.23) |
|  |  | Control group | -6.585 | <0.001 | 29.42 (20.94 to 37.89) |
|  | Week 39 -first visit | Experimental group | -10.954 | <0.001 | 66.82 (55.07 to 78.57) |
|  |  | Control group | 8.365 | <0.001 | 37.47 (28.65 to 46.28) |
|  | Week 52 -first visit | Experimental group | 12.391 | <0.001 | 70.66 (59.43 to 81.89) |
|  |  | Control group | 8.827 | <0.001 | 40.57 (31.52 to 49.62) |
|  | Week 78 -first visit | Experimental group | 12.097 | <0.001 | 63.32 (53.02 to 73.63) |
|  |  | Control group | 7.717 | <0.001 | 36.47 (27.17 to 45.78) |
|  | Week 104 -first visit | Experimental group | -9.137 | <0.001 | 55.60 (44.76 to 66.43) |
|  |  | Control group | -6.305 | <0.001 | 34.45 (23.94 to 44.95) |
| **CAT** | Week 13 -first visit | Experimental group | -9.374 | <0.001 | -3.80 (-4.51 to -3.10) |
|  |  | Control group | -7.275 | <0.001 | -2.50 (-3.15 to -1.84) |
|  | Week 26 -first visit | Experimental group | -9.045 | <0.001 | -4.22 (-5.02 to -3.41) |
|  |  | Control group | -6.892 | <0.001 | -2.78 (-3.54 to -2.01) |
|  | Week 39 -first visit | Experimental group | -9.690 | <0.001 | -5.23 (-6.12 to -4.35) |
|  |  | Control group | -5.958 | <0.001 | -2.87 (-3.74 to -2.00) |
|  | Week 52 -first visit | Experimental group | -9.466 | <0.001 | -5.50 (-6.46 to -4.53) |
|  |  | Control group | -4.856 | <0.001 | -2.31 (-3.17 to -1.46) |
|  | Week 78 -first visit | Experimental group | -9.289 | <0.001 | -5.60 (-6.61 to -4.59) |
|  |  | Control group | -5.166 | <0.001 | -2.83 (-3.81 to -1.85) |
|  | Week 104 -first visit | Experimental group | -8.664 | <0.001 | -5.21 (-6.22 to -4.19) |
|  |  | Control group | -5.880 | <0.001 | -3.38 (-4.38 to -2.37) |

**Abbreviations:** mMRC, modified Medical Research Council dyspnea scale; 6MWT, 6-minute walking test; CAT, COPD Assessment Test; MD, mean difference; CI, confidence interval.
